# Emerging role of lipid metabolism alterations in Cancer stem cells

**DOI:** 10.1186/s13046-018-0784-5

**Published:** 2018-06-15

**Authors:** Mei Yi, Junjun Li, Shengnan Chen, Jing Cai, Yuanyuan Ban, Qian Peng, Ying Zhou, Zhaoyang Zeng, Shuping Peng, Xiaoling Li, Wei Xiong, Guiyuan Li, Bo Xiang

**Affiliations:** 1 Hunan Provincial Cancer Hospital and Cancer Hospital Affiliated to Xiangya Medical School, The Central South University, Changsha, 410013 Hunan China; 2 Department of Dermatology, Xiangya hospital of Central South University, Changsha, 410008 China; 3 Cancer Research Institute, Xiangya School of Medicine, Central South University, Changsha, 410078 China

**Keywords:** Cancer stem cells, Lipid metabolism, Metabolomics, Lipid droplets, Lipid desaturation, de novo lipogenesis, Fatty acid oxidation

## Abstract

**Background:**

Cancer stem cells (CSCs) or tumor-initiating cells (TICs) represent a small population of cancer cells with self-renewal and tumor-initiating properties. Unlike the bulk of tumor cells, CSCs or TICs are refractory to traditional therapy and are responsible for relapse or disease recurrence in cancer patients. Stem cells have distinct metabolic properties compared to differentiated cells, and metabolic rewiring contributes to self-renewal and stemness maintenance in CSCs.

**Main body:**

Recent advances in metabolomic detection, particularly in hyperspectral-stimulated raman scattering microscopy, have expanded our knowledge of the contribution of lipid metabolism to the generation and maintenance of CSCs. Alterations in lipid uptake, de novo lipogenesis, lipid droplets, lipid desaturation, and fatty acid oxidation are all clearly implicated in CSCs regulation. Alterations on lipid metabolism not only satisfies the energy demands and biomass production of CSCs, but also contributes to the activation of several important oncogenic signaling pathways, including Wnt/β-catenin and Hippo/YAP signaling. In this review, we summarize the current progress in this attractive field and describe some recent therapeutic agents specifically targeting CSCs based on their modulation of lipid metabolism.

**Conclusion:**

Increased reliance on lipid metabolism makes it a promising therapeutic strategy to eliminate CSCs. Targeting key players of fatty acids metabolism shows promising to anti-CSCs and tumor prevention effects.

## Background

Cancer stem cells (CSCs) or tumor initiating cells (TICs) are small subpopulations (0.001–0.1%) of cancer cells that may account for cancer initiation, metastasis, therapy resistance, and recurrence [1, 2]. CSCs exhibit self-renewal and tumor-initiating properties and are able to recapitulate the bulk hierarchy of cancer tissues [3]. Evidence suggests that epithelial-mesenchymal transition (EMT) is associated with stemness acquisition. Cancer cells after EMT usually exhibit stem cell-like characteristics, and thus CSCs are also believed to act as metastasis-initiating cells [4–8]. The origin of CSCs is still under debate. It has been proposed that CSCs may arise from normal stem cells or tissue progenitor cells as a result of stochastic genetic mutations and epigenetic alterations [9]. Nevertheless, some evidence suggests that differentiated cells might also stochastically dedifferentiate into a more primitive state with tumor-initiating potential [10–12]. Kelly et al. demonstrated that large numbers of leukemia-initiating cells (LICs) can recapitulate the bulk hierarchy of cancer in genetically compatible models [11]. They claimed that the rarity of TICs in xenotransplant experiments is mainly a result of the limited ability of human tumor cells to adapt and survive in an alien (mouse) milieu. However, the hierarchical and stochastic models are not mutual exclusive. Though not all cancers follow the hierarchical model [13, 14], the presence of CSCs is clearly demonstrated in various cancer types using distinct cell surface markers and enzymatic assays, including leukemia [15], breast cancer [16], glioblastoma [17], colorectal cancer [18–20], pancreatic cancer [21–23], liver cancer [24], lung cancer [25], and ovarian cancer [26, 27]. For example, in acute myeloid leukemia (AML), CSCs populations are CD34(+) CD38(−) [15]. In breast cancer, CSCs were identified as CD44(+) CD24(−/low) lineage [16] or ALDH1-positive [28] populations. In glioma, the CD133(+) or CD44^(high)^/Id1^(high)^ fractions are recognized as CSCs [17, 29]. Overexpression of CD44 variant isoform (CD44v), which is mainly generated by ESRP1 and ESRP2 mediated alternative splicing on CD44 mRNA [30], is observed in various CSCs [31] High level of CD44v8–10 protects CSCs from reactive oxygen species (ROS), which is known to play a “double-edged sword” role in cancer development [31].

## Autophagy, Ferroptosis and redox regulation in CSCs

It remains a great challenge to eliminate CSCs and improve the survival of patients, because CSCs are typically quiescent and resistant to conventional radio- and chemotherapy [32, 33]. It was believed that CSCs largely contribute to formation of clinically undetectable minimal residual disease(MRD) after conventional anti-tumor therapies, and therefore are implicated in disease persistence or relapse. Alterations in cellular bioenergetics impart CSCs in MRD to develop adaptive or acquired resistance to anti-tumor therapy, thus leading to tumor recurrence [34]. For example, comprehensive transcriptomic and metabolic analyses of oncogene ablation-resistant pancreatic cancer cells possessing CSCs characteristics revealed enhanced mitochondrial respiration but diminished dependence on the Warburg effect, as well as increased autophagy and lysosome activity, suggesting metabolic alterations and active autophagy are critical features of CSCs [35]. Autophagy primarily acts as a lysosomal dependent metabolic-recycling mechanism which is important for cell survival in stress [36–38]. It has been considered that autophagy may exert a anticarcinogenic role in early stage of cancer development by safeguarding against genomic instability through the clearance the old and dysfunctional mitochondria and protein aggregates [36, 39]. Furthermore, autophagy may exert tumor suppressive function through destabilizing the transcription factor nuclear factor erythroid 2-related factor 2 (NRF2), which imparts tumor cells with resistance to redox stress [40]. Nevertheless, active autophagy is recognized as one of the hallmarkers of cancer [41]. In established cancers, constitutive activation of autophagy contributes to accquired therapeutic resistance. For example, active autophagy protects glioblastoma multiforme (GBM) cells from the unfavorable tumor microenvironment characterized by hyper-oxidative, hypoxic, nutrient-poor conditions [42]. Further more, compelling evidence suggests that autophagic-lysosomal pathway largely contributes to generation, maintenance and differentiation of CSCs [43]. Many studies have shown that CSCs frequently have higher basal level of autophagy than that of non-stem cancer cells. Active autophagy help CSCs to rapidly respond to metabolic stress to maintain their energetic balance. For example, the fusion of lipid droplets with autophagosomes, a process named lipophagy, confer a survival advantage on oncogene ablation-resistant pancreatic cancer cells through increase of fatty acid β-oxidation [35]. A number of studies have shown either chemical or genetical blockade of autophagy impairs self-renewal and tumorigenicity of CSCs [44–46]. Recent studies revealed that some of “conventional” agents used in non-cancerous diseases treatment exert antitumor therapeutic effects by modulating autophagic pathway, suggesting that drug re-positioning targeting autophagy may be a promising therapeutic strategies for human malignancies [40].

Ferroptosis is recognized as an iron-dependent form of nonapoptotic cell death implicated in various human diseases, including ischemic tissue damage and human malignant diseases [47]. Recent study unveiled the crucial role of autophagy in ferroptosis. Pharmacological induction of ferroptosis leads to lysosomal degradation of cellular iron storage proteins ferritin and ferritinophagy cargo receptor NCOA4 in an autophagy dependent manner (a process known as ferritinophagy), suggesting the close relationship between ferroptosis and autophagic cell death [48]. Recently, a synthetic derivative of natural product salinomycin named as ironomycin sequesters lysosomal iron and induces ferroptosis, showing a selective antitumor activity against breast CSCs in vitro and in vivo [49]. In addition, ferroptosis is triggered by iron-dependent excessive lipid hydroperoxides accumulation due to insufficient antioxidant glutathione (GSH) level, the cystine/glutamate antiporter system x(c)(−) is likely to be involved [50]. System x(c)(−) is composed of a light chain, xCT, and a heavy chain, 4F2 heavy chain (4F2hc) [50]. Upregulation of xCT contributes to drug resistance in pancreatic cancers [51]. Up-regulation of xCT also has been demonstrated in other cancers in including lymphomas [52], and gliomas [53, 54]. CD44v has recently been shown to involved in the scavenging of ROS via the stabilization of xCT protein at the cellular membrane, thus activation of CD44v-xCT-GSH axis play a crucial role in redox regulation of CSCs and is likely contribute to the relapse and distant metastasis after repeated radiation therapy [55]. Remarkably, chemotherapy is able to induce ectopic expression of CD44v, which is evidenced in osteosarcoma and hepatic cancer cells of the Li-Fraumeni patient [56]. This is probably due to the selective clonal amplification of undetectable number of CD44v8–10-positive CSCs under the pressure of excessive ROS after radiation and chemotherapy.

## Metabolic alterations in human Cancer

Cancer cells exhibit a distinct metabolic profile as compared to it’s normal counterparts. Due to the rapid tumor cells proliferation and inadequate blood vessels formation, tumor microenviroments are characterized by hypoxic, hyper-oxidative, acidic and nutrients-poor conditions, therefore cancer cells must adapt it’s cellular bioenergetics efficiently to deal with this kind of unfavorable microenvironments, a process named metabolic reprogramming. Metabolic reprogramming is essential to sustain cancer cells proliferation and survival when the oncogenic signaling is blocked [35, 57]. Most human cancers show constitutive aerobic glycolysis even in oxygen-rich conditions, a phenomenon called Warburg effect [58, 59]. This kind of metabolic rewiring not only satisfies the energy demands for continuous proliferation, but also provides plenty of building blocks for cellular compartments. Metabolic regulation of stemness is increasingly recognized as fundamental in the control of stem cell fate. In contrast to most differentiated cells, pluripotent stem cells (PSCs) rely primarily on aerobic glycolysis rather than mitochondrial oxidative phosphorylation(OXPHOS) to minimize ROS production, which impairs self-renewal ability [60, 61]. Reduced mitochondrial respiration in quiescent hematopoietic stem cells (HSCs) prevent oxidative damage from ROS, enabling long-term survival because HSCs are sensitive to ROS [62]. Aerobic glycolysis also contributes to acquisition of stemness in CSCs. It has been demonstrated that poorly differentiated cancers show much higher glucose uptake than differentiated cancers, suggesting that a high glycolytic flux in tumor tissues mainly results from a blockade of CSCs differentiation [63]. Conversely, activation of mitochondrial metabolism leads to loss of pluripotent potential and induction of differentiation in P19 embryonal carcinoma stem cells [64]. Recently, Peng et al. demonstrated that breast cancer stem cells (BCSCs) have high levels of pyruvate dehydrogenase kinase 1 (PDK1), which inhibits mitochondrial OXPHOS. Depletion of PDK1 significantly diminishes ALDH1-positive BCSCs, which leads to decreased sphere-formation ability [65], suggesting targeting aerobic glycolysis may be usefull to eliminating CSCs. However, to date, attempts to inhibit glycolysis as cancer therapy remain unsatisfactory [66], which is mainly due to CSCs are very heterogeneous and may thus have divergent metabolic landscapes [67]. In addition to glucose, some cancer cells also use glutamine heavily [68]. However, little is known about the role of glutaminolysis in stem cell homeostasis.

Unlike HSCs, normal neuro stem cells(NSCs) show low levels of glycolysis [69], suggesting that the metabolic phenotype of pluripotent cells is highly plastic and strongly influenced by tissue microenviroments and nutrient availability [70]. A growing body of evidences indicates that CSCs/TICs are more dependent on oxidative metabolism than glycolysis [61, 71]. For example, oncogene ablation resistant pancreatic cells with feature of CSCs strongly rely on mitochondrial respiration rather than Warburg effect [35]. CSCs from ovarian cancer primarily rely on fatty acid β-oxidation (FAO) and are resistant to glucose deprivation [72].

## Metabolic and redox cues of Phenoconversion in CSCs

As mentioned above, metabolic cues play a central role in cell fate determination. A growing body of literatures indicates that metabolism reprogramming and CSCs properties are two highly entwined processes during tumor development [73]. On one hand, chronic metabolic stress in premalignant environments may drive the phenoconversion of non-stem cancer cells to a stem-like state in a Wnt-dependent manner [74]. In addition, chronic oxidative stress at non-cytotoxic doses promotes neoplastic transformation and stem cell characteristics in kidney epithelial cells [75], suggesting that ROS may act as a “double-edged sword” in the acquisition of stem cell characteristics in a dose-dependent manner. On the other hand, impairment of mitochondrial metabolism via inhibition of complex I or loss of mitochondrial DNA leads to genetic inactivation of p53 and to a glycolytic switch in neural progenitor/stem cells (NPCs), which result in genomic instability and glioma formation and support the notion that metabolic stress triggers the conversion of normal NSCs to a glioma-initiating NSCs [69]. In the established tumor tissues, tumor cells continually undergo persistent and high level of oxidative stress [76]. In terms of the survival against excessive degree of redox stress, CSCs must adapt it’s cellular bioenergetics efficiently to this kind of unfavorable conditions, a NRF2-dependent anti-ROS signal pathway may be involved [34] . Activation of NRF2 promotes tumor cells resistant to redox stress, whereas inactivation of NRF2 with the flavonoid chrysin effectively sensitizes BEL-7402/ADM tumor cells to doxorubicin by downregulating the PI3K/Akt and ERK pathways [77]. Redox balance may contribute to autophagy associated drug resistance, that why NRF2 inhibitors suppress cancer stemness and sensitize GBM cells to temozolomide(TMZ), an alkylating agent for GBM and anaplastic astrocytoma treatment, which induces autophagy and subsequent therapeutic resistance [42, 78]. Recently, Yoshida et al. demonstrated that CD44v but not the standard CD44, promotes proteasome degradation of c-Myc protein via suppressing redox stress-induced Wnt activation [31]. High amount of CD44v8–10 cooperate with Fbw7, a well-defined ubiquitin ligase of c-myc protein [79], precisely regulate the proliferation and dormancy cycle of CSCs through modulating c-myc protein stability at the invasive front [31, 80].

Studies unveiled that glioma CSCs reside in either perivascular niche or perinecrotic microenviroment [81, 82]. In the perivascular niche, glioma CSCs interact closely with endothelial cells which secrete factors to maintain the self-renewal of CSCs [81]. It has been demonstrated that the CD44 ligand osteopontin enriched in perivascular niche promotes glioma CSCs-like phenotypes and radiation resistance. These effects were mediated by HIF-2α in a cooperative manner with γ-secretase generated CD44 intracellular domain [83]. On the other hand, a hypoxic and perinecrotic microenviroment, which known to stimulate glycolysis and induce autophagy, promote acquisition of a stem-like state and increase the CSCs population through stabilization of both HIF1 and HIF2 [82, 84, 85]. It has been shown that two of pluripotency transcription factors, OCT4 and c-Myc, were directly activated by HIF-2α [86, 87].

## Alterations in lipid metabolism in CSCs

Though the bulk tumor cells and CSCs share some common metabolic features as compared to normal cells, it has been proposed that the metabolic state of TICs/CSCs subtlely differs from that of non- stem cancer cells [88]. Fatty acids metabolism not only supports energy production but also plays an important role in biosynthetic pathways and redox homeostasis. Recent advances in proteomics and metabolomics have deepened our knowledge of the role of fatty acids metabolism in determining CSCs fate [89–91]. For example, Chen et al. described that NANOG, a master factor in controlling stem cell fate, stimulates the generation of stem-like TICs and hepatocellular carcinoma (HCC) oncogenesis via metabolic reprogramming from OXPHOS to fatty acids oxidation [60], suggesting lipid metabolism is fundamental for NANOG positive CSCs.

**Fig. 1:**
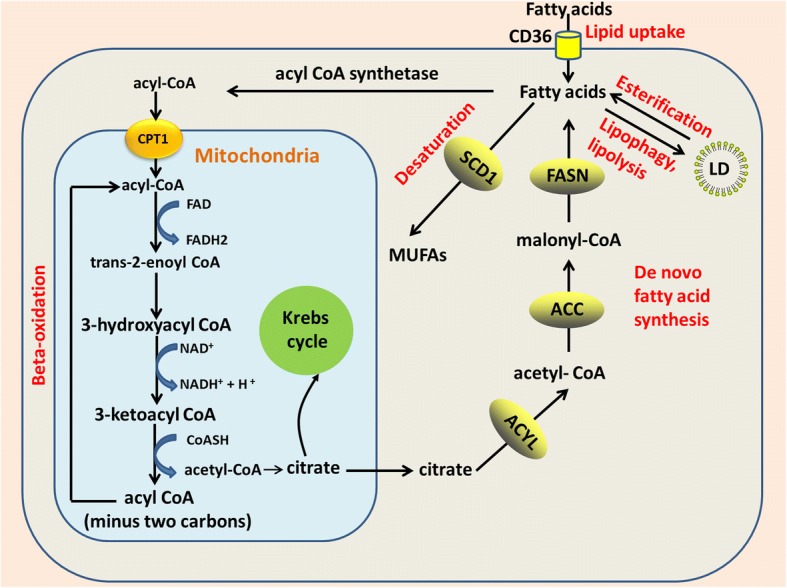
Alterations in lipid metabolism in CSCs. Both lipid catabolism and anabolism alterations contribute to stemness acquisition in CSCs, including lipid uptake, de novo lipogenesis, lipid desaturation, lipolysis, lipophagy, and FAO. Extracellular FFAs are transported into cells via CD36 and then reused via β-oxidation in mitochondria to release acetyl-CoA. Acetyl-CoA is converted to citrate by citrate synthase and then enters the Krebs cycle for complete oxidation. Alternatively, de novo fatty acids synthesis starts with acetyl-CoA and builds up by the addition of two-carbon units. In addition to lipid catabolism, fatty acids are esterified to glycerol and then triglycerides are stored in lipid droplets. Breakdown of lipids droplets via lipolysis or lipophagy enables stored energy mobilization to the mitochondria. Additionally, saturated fatty acids are desaturated into mono-unsaturated fatty acids by SCD1. Alterations in lipid metabolism not only satisfy energy demands for CSCs proliferation, but also provide essential components for biosynthetic pathways and redox homeostasis. ACC, acetyl-CoA carboxylase; ACLY, ATP citrate lyase; FASN, fatty acid synthase; CD36, cluster of differentiation 36; FAs, fatty acids; MUFAs, mono-unsaturated fatty acids; SCD1, stearoyl-CoA desaturase 1; CPT1, carnitine palmitoyltransferase 1; TCA cycle, tricarboxylic acid cycle; FAO, fatty acid oxidation; and LD, lipid droplet

Lipids are essential components of cell and organelle membranes, and fatty acids are required for proliferation of the bulk tumor mass and also for CSCs maintenance [89, 92, 93]. There is a strong contribution from the lipid metabolism, whereas the role of glycolysis in CSCs maintenance may be more tumor-specific. For example, glioma stem cells (GSCs) use less glycolysis than differentiated glioma cells but maintain higher levels of ATP production [94]. Further more, the glycolytic intermediates could be used by CSCs for de novo lipogenesis to increase self-renewal growth [66], suggesting different metabolic pathway could be well coordinated in CSCs to maximize the benefits. Both lipid catabolism and anabolism alterations are associated with acquisition of stemness during cancer development (Fig. 1). For example, BCSCs exhibit elevated long-chain FAO metabolites compared to non-stem cancer cells. Moreover, inhibition of FAO by etomoxir markedly decreases viability and tumorsphere-forming potential of BCSCs but exert little effect on non-stem cancer cells, suggesting that FAO is critical to self-renewal of BCSCs [89].

### Lipid droplets in CSCs

**Fig. 2:**
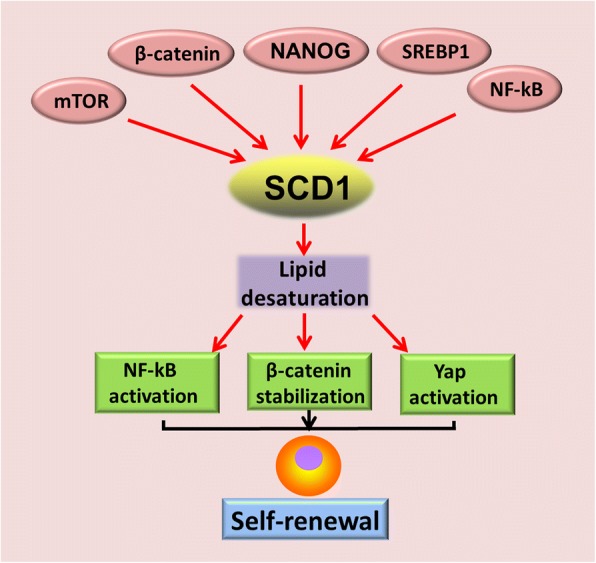
NANOG mediated metabolic reprogramming contributes to CSCs self-renewal and chemoresistance. NANOG binding on FAO genes(Acadvl, Echs1, and Acads) promoters stimulates it’s transcription but exerts opposite effect on OXPHOS genes(Cox6a2 and Cox15) transcription, leading to metabolic switch from OXPHOS to FAO and less ROS production in CSCs/TICs. NANOG also promotes lipid desaturation via up-regulating SCD1 expression. OXPHOS, oxidative phosphorylation; FAO, fatty acid oxidation

Lipid droplets (LDs) are intracellular spherical organelles surrounded by a single layer of phospholipids that store lipids [95]. Cancer cells have more LDs compared to normal cells [95]. During metabolic stress resulting from blocked glycolysis, free fatty acids (FFAs) from LDs sustain ATP production though FAO (Fig. 1). Breakdown of LDs in an autophagy-dependent manner, a selective autophagy named lipophagy, enables FFAs mobilization to the mitochondria (Fig. 1), which is pivotal for survival when metabolic restrictions in cancer cells are induced by oncogenic signaling blockade [57, 96]. LDs also protect lipid from peroxidation, as toxic lipid peroxides trigger ferroptosis [47]. In addition, lipid quantification in prostate cancer is associated with the tumor stage, thus serving as a quantitative marker for disease diagnosis [97]. LDs formation is induced by hypoxia via HIF1- and HIF2-mediated repression of carnitine palmitoyltransferase 1A (CPT1A), a key enzyme in involved in mitochondrial FAO [98] (Fig. 2). In addition to de novo lipogenesis, increase in extracellular lipid uptake also contributes to LDs accumulation and tumor initiation capacity in CSCs [99] (Fig. 1).

CSCs display more LDs compared to the bulk cancer cells in several cancer types. For example, Tirinato et al. demonstrated that colorectal cancer stem cells (CRCSCs) exhibit higher lipid levels compared to normal epithelial colon cells (NECCs), colon carcinoma cells (CCCs), and sphere-derived adherent cancer cells (SDACs). The authors showed that CRCSCs have higher LDs content than SDACs and CCCs, whereas NECCs exhibit the lowest LDs content. Interestingly, lipid content in colorectal cancer cells directly correlates with CD133 and Wnt pathway activity. Furthermore, CRCSCs with high LDs content exhibit higher clonogenic and tumorigenic potential than LD^low^ CRCSCs [100]. Similarly, ovarian cancer stem cells (OCSCs) (ALDH^+^/CD133^+^) isolated from the COV362 cell line have higher LDs content than ALDH^−^/CD133^−^ cancer cells [90]. Elevated LDs content in CSCs not only provide an alternative energy source when glycolysis is blocked, but also protect fatty acids from harmful peroxidation in the stem cell niche, thus enabling stem cell proliferation [101]. Whereas inhibition of phospholipase A2 leads to reduction in LDs and triggers apoptosis in cancer cells [57].

### De novo lipid biosynthesis in CSCs

A metabolic hallmark of cancer is the increase in de novo lipogenesis [102] (Fig. 1). Unlike most non-malignant cells, cancer cells are highly dependent on de novo lipogenesis to satisfy energy demands because of the limited availability of dietary lipids. CSCs may siphon glycolytic metabolic intermediates into de novo lipid biosynthesis to increase self-renewal growth [66]. Yasumoto et al. reported that both ^14^[C]-glucose and ^14^[C]-acetate incorporation into lipids is more pronounced in GSCs, indicating that de novo lipogenesis is more active in these cells than in differentiated bulk glioma cells [103]. Intriguingly, fatty acid synthase (FASN), a key lipogenic enzyme, is overexpressed in patients-derived GSCs but is dramatically decreased upon serum-induced differentiation, suggesting that enhanced de novo lipogenesis contributes to maintain the undifferentiated status of GSCs. Inhibition of lipogenesis in GSCs by pharmacologically targeting FASN with cerulenin significantly reduces stemness marker (SOX2, nestin, CD133, and FABP7) expression levels, invasiveness, and sphere formation in GSCs, whereas glial fibrillary acidic protein GFAP levels are increased. Using proteomic and metabolomic analyses, Brandi et al. demonstrated that pancreatic CSCs have higher levels of glycolysis and increased de novo lipogenesis activity compared to bulk parental cancer cells, but reduced mitochondrial OXPHOS levels. The authors showed that FASN is overexpressed and is more sensitive to inhibition by cerulenin in Panc1 CSCs than in the parental non-stem cancer cells. [91]. BCSCs (CD24^−^ CD44^+^ ESA^+^) isolated from MCF10DCIS.com cells, which give rise to ductal carcinoma in situ, exhibit higher expression levels of lipogenic genes such as ATP citrate lyase (ACLY), acetyl CoA carboxylase 1 (ACC1), and FASN compared to non-stem cancer cells(Fig. 1). Ectopic expression of sterol regulatory element-binding protein-1 (SREBP1) gene, a master regulator of lipogenesis, upregulates downstream lipogenic genes (ACLY, ACC1, and FASN) concomitant with increased lipogenesis, growth, and mammospheres formation in MCF10A stem-like cells. Upregulation of these lipogenic genes promotes cell viability and proliferation in MCF10A stem-like cells [104]. ACC converts acetyl-CoA to malonyl-CoA during lipid biosynthesis. Inhibition of ACC by soraphen A also notably decreases the number of ALDH1^+^-CSCs-like cells and impairs mammosphere formation in MCF-7 cells [66].

### Lipid desaturation in CSCs

Lipid unsaturation is essential in breast, colon, and prostate cancer cells [105, 106]. Administration of unsaturated fatty acids by gavage to BALB/c mice pre-inoculated with colorectal cancer cells amplify CD133(+) subpopulations, induces stemness and promotes tumor formation and metastasis [107]. Most mono-unsaturated fatty acids (MUFAs) are the catalytic product of stearoyl-CoA desaturase (SCD) (Fig. 1), a rate-limiting enzyme, that adds a cis-double bond at the delta 9 position in acyl-CoA chains [108]. It has been shown that overexpression of SCDs promotes cancer cells proliferation and inhibits cell death [105, 106, 109].

Lipid unsaturation was recently recognized to be a unique metabolic biomarker for ovarian CSCs [90]. Ovarian CSCs harbor more unsaturated lipid-containing LDs than non-stem cancer cells, implying that CSCs rely much more on unsaturated lipids than bulk tumor cells. Blocking lipid desaturase decreases ovarian CSCs marker expression and prevents tumor initiation in vivo [90], which is consistent with the pilot study that SCD1 acts as a stemness regulator in breastcancer [109]. Importantly, these observations indicate that lipid desaturase may be an ideal target for tumor prevention in cancers from various tissue origins.

There are currently several possible mechanisms underlying CSCs regulation by lipd desaturation. Lipids are essential components of cellular membranes, the fluidity of which is determined by the degree of lipid unsaturation. Membrane mechanical properties are critical in cell division, migration, and signal transduction [110]. Reducing membrane fluidity exerts an inhibitory effect on metastatic capacity and stem cell-like properties of breast cancer cells [111]. Increase in polyunsaturated fatty acids prevent lipotoxicity of saturated fatty acid (SFA) to the membrane system, which impair membrane fluidity [112]. Palmitate, a lipotoxic metabolite mainly derived from SFA, drives solid-like domain separation from the ER membrane and thus reduces membrane fluidity [113]. Decrease in palmitic acyl (C16:0)-containing glycerophospholipids promotes HCC cell proliferation and invasiveness, highlighting that excessive palmitic acid impairs HCC development [114]. Compared to non-CSCs, CSCs have lower cell membrane tension and exhibit significant shape deformation in response to stimuli. Increase in membrane tension by immersing CSCs in hypotonic medium leads to a decrease in polarized CSCs [115]. Because cell polarity is essential for asymmetric division and migration [116, 117], CSCs would benefit from increased fatty acid unsaturation in lowering membrane tension and preventing symmetric division or loss of pluripotency. It’s worthy to note that lipotoxic metabolites, including palmitate and stearate, are the preferred substrates of SCDs [108], suggesting SCDs may be crucial to maintain membrane fluidity of CSCs.

Recent studies have further deepened our understanding of how lipid desaturation interplays with oncogenic signaling pathways to generate CSCs. It has been demonstrated that NF-kB signaling activation and ALDH1A1 promotes lipid desaturation [90] (Fig. 2). Reciprocally, inhibition of desaturases with CAY 10566 and SC-26196 dramatically represses NF-kB transcriptional activity and ovarian CSCs characteristics, though the detailed mechanisms underlying NF-kB activation by SCD1 or unsaturated fatty acids still remain unclear. MUFAs produced by SCDs also amplify Wnt signaling via stabilization of β-catenin in rodent hepatic stellate cells (HSCs) and mouse liver TICs [118] (Fig. 2). Furthermore, MUFAs increase cytosolic levels of nuclear import of elav-like protein 1 (HuR), thus promoting HuR-mediated stabilization of Lrp5 and Lrp6 mRNAs [118]. The third oncogenic signaling link to lipid desaturation in CSCs generation is the Hippo/YAP signaling pathway. SCD1 activity promotes nuclear accumulation of YAP and increases transcriptional activity in lung adenocarcinoma CSCs in a Wnt-dependent manner, which is evidenced by Wnt3a rescuing YAP protein from SCD1 inhibition(Fig. 2) [119]. Co-expression of SCD1 with β-catenin and YAP/TAZ transcriptional target birc5 predicts unfavorable clinical outcomes in lung adenocarcinoma patients [119]. In addition to directly promoting stemness in CSCs, unsaturated fatty acids also stimulate mesenchymal stem cells to increase secretion of angiogenic factors such as interleukin-6, VEGF, and nitric oxide [120], which play a crucial role in angiogenesis and metastasis in human cancers.

### Elevated lipolysis and extracellular lipid uptake sustain CSCs

FFAs produced by host cell lipolysis also fuel tumor growth [121]. Recently, Singh et al. demonstrated that blocking lipolysis in the digestive system of adult *Drosophila melanogaster* selectively induces necrotic death in normal and transformed stem cells without affecting differentiated cells [122]. Melanosphere-derived CSCs have increased lipid uptake when compared with differentiating melanosphere-derived cells [123]. Leukemic stem cells (LSCs) residing in gonadal adipose tissue (GAT), which act as a LSC niche to support LSC metabolism, trigger lipolysis to release FFAs through secretion of pro-inflammatory cytokines such as TNF-α, IL-1α, IL-1β, and CSF2. These FFAs are transported into LSCs via CD36(Fig. 1), a fatty acid transporter enriched in a sub-population of LSCs, and then reused via β-oxidation in LSC mitochondria to support LSC survival and evade chemotherapy. Loss of CD36 reduces homing of LSCs to GAT and leukemic burden in mice [124]. Enrichment of CD36 was also observed in glioma CSCs. Uptake of oxidized phospholipids such as oxLDL, a natural ligand of CD36, drives glioma CSCs proliferation but exerts no effect on differentiated glioma cells [125]. In addition to affecting proliferation of CSCs, uptake of palmitic acid via CD36 also specifically activates the metastatic potential of CD44^bright^ oral squamous cell carcinoma (OSCC) metastasis-initiating cells [126], highlighting the central role of lipids uptake in fueling tumor metastasis.

### Elevated FAO fuels CSCs

**Fig. 3:**
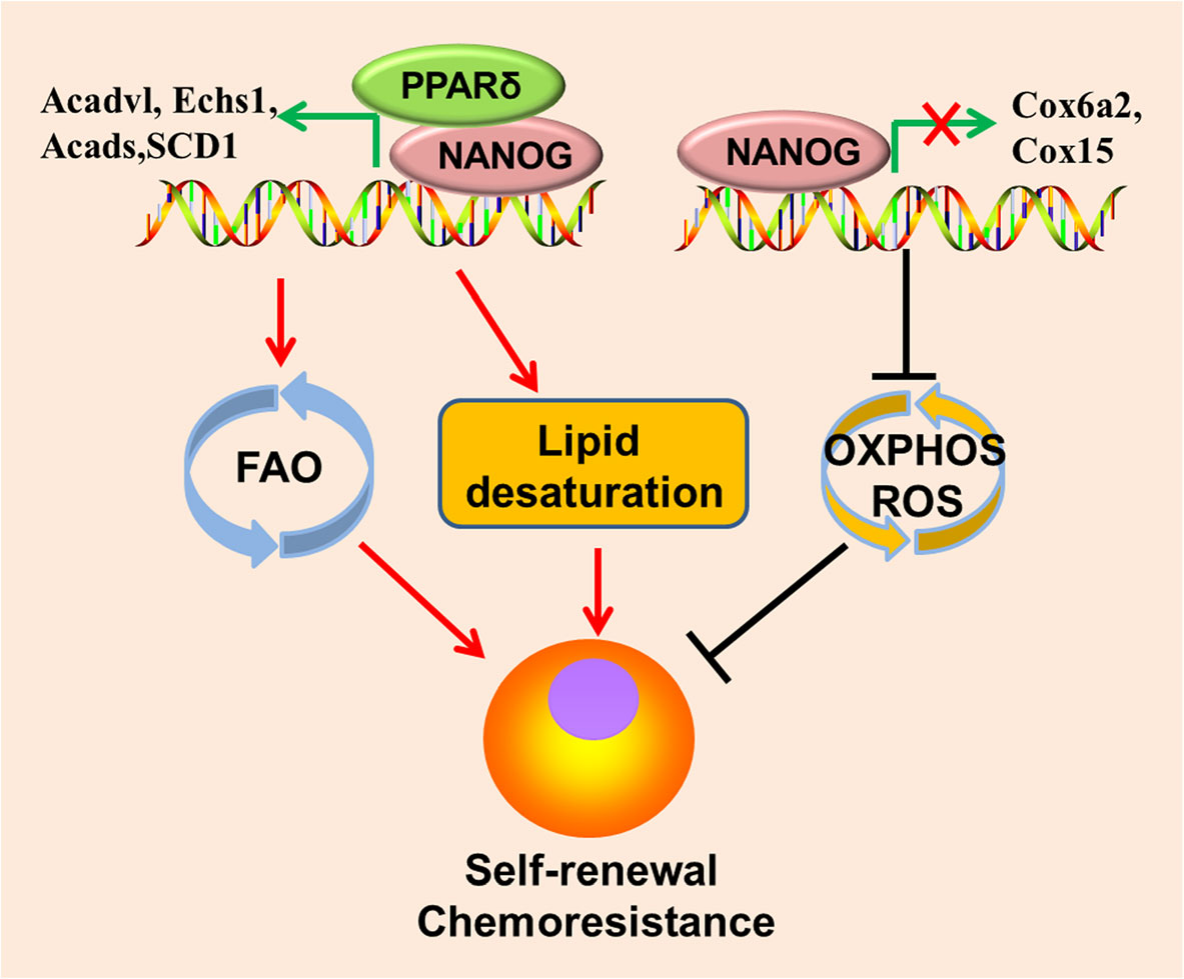
Regulation of SREBP1 and lipid metabolism by oncogenic signaling in CSCs. Oncogenic PI3K (H1047R)- and K-Ras (G12 V) activates SREBP1 and SREBP2 to support de novo lipid synthesis and cell growth. The mTOR signaling regulates SREBP1 level through both transcriptional or translational mechanisms. Activation of PI3K.AKT/mTOR signaling pathway or FGFR3 leads to stabilization of SREBP1 protein and promotes SREBP1 translocation to nucleus. Mitotic kinase Cdk1 and Plk1 physically interact with nuclear SREBP1 protein. Sequentially phosphorylation of SREBP1 by Cdk1 and Plk1 blocks binding between the ubiquitin ligase Fbw7 and SREBP1 and attenuates SREBP1 degradation. Upon EGFR signaling activation, the nuclear form of PKM2 physically interacts with SREBP1, activating SREBP target gene expression and lipid biosynthesis

Oncogenic K-Ras mutation contributes to CSCs activation in colorectal cancer tumorigenesis, increased FAO may be involved [127]. Oncogenic K-ras (G12D) activation stimulates mitochondrial FAO to support metabolism and drive non-small cell lung cancer (NSCLC) development via up-regulating autophagy [128]. MYC-driven triple-negative breast cancer (TNBC) has an increased reliance on FAO for uncontrolled tumor growth [129]. Furthermore, mitochondrial FAO also drives triple negative breast cancer cells(TNBC) metastasis [130]. A recent study unveiled that NANOG stimulates mitochondrial FAO gene expression but represses mitochondrial OXPHOS gene expression [60] (Fig. 3). Metabolic reprogramming from OXPHOS to FAO is critical for NANOG-mediated HCC TIC generation [60]. Inhibition of FAO impairs TIC self-renewal and tumorigenicity and sensitizes TICs to sorafenib, which is a broadly used chemotherapy drug against HCC.

Mitochondrial FAO plays an important role in satisfying energy requirements in TICs (Fig. 1). Increased FAO supports CSCs survival when glucose metabolism becomes limiting [131, 132]. Increase in FAO is critical to inflammatory signaling-mediated CSCs generation. For example, inhibition of FAO blocks BCSCs self-renewal and increases its chemo-sensitivity [89]. Activation of Src oncoprotein is also associated with CSCs generation [133]. FAO plays a crucial role in Src oncoprotein activation through autophosphorylation at Y419 in TNBC [134]. LSCs lacking CPT1A, a rate-controlling enzyme in FAO, are refractory to avocatin B, a lipid derived from avocado fruit that selectively kills AML stem cells with little effect on its normal counterpart [135], highlighting the importance of FAO in the establishment of chemo-resistance.

Mitochondrial FAO also benefits stem cells via several different mechanisms. First, FAO reduces ROS production, which is harmful to stem cells [131], that why disrupting their redox defense capability exerts therapeutic effect against CSCs [136]. Second, mitochondrial FAO is essential for pluripotency maintenance in HSCs and NSCs via controlling the asymmetric division in HSCs [137, 138]. Reduced FAO flux potentiates NSCs symmetric differentiating divisions at the expense of self-renewal [139]. Third, FAO pathway activation by peroxisome proliferator-activated receptor contributes to Tie2+ HSC expansion through induction of mitophagy [140]. In addition to maintaining the redox balance of CSCs, mitochondrial FAO is also important for epigenetic regulation of gene transcription, because acetyl coenzyme A, an intermediate product of FAO, is required for histone acetylation by the histone acetyltransferase p300 [141].

## Key modulators of lipid metabolism in CSCs

### NANOG

NANOG is a key transcription factor implicated in pluripotency maintenance and self-renewal in ES cells. NANOG is expressed in various human cancers and is associated with poor prognosis [142–144]. Re-activation of NANOG in CSCs is critical for stemness maintenance and self-renewal [145–147], in which it orchestrates mitochondrial metabolic reprogramming [60]. ChIP-seq revealed that NANOG binds to mitochondrial OXPHOS gene (Cox6a2 and Cox15) promoters and represses those genes transcription. In contrast, NANOG binds to FAO genes (Acadvl, Echs1, and Acads) promoters and stimulates it’s transcription, a cooperative interaction with peroxisome proliferator-activated receptor δ(PPARδ) involved(Fig. 3). Thus, overexpression of NANOG represses mitochondrial respiratory activity and ROS production, but stimulates FAO to favor HCC TIC self-renewal and chemo-resistance, whereas silencing NANOG has opposite effects [60]. Either re-expression of OXPHOS genes (Cox6a2 or Cox15) or silencing FAO genes (Echs1 and Acadvl) leads to impairment of TIC self-renewal, suggesting that reprogramming the metabolism from mitochondrial OXPHOS to FAO by NANOG is an intrinsic character of CSCs, not a conditional adaptation. Interestingly, NANOG reduces long-chain FA levels but upregulates SCD1 expression in HCC TICs [60] (Fig. 3). Inhibition of SCD by PluriSin#1, a small-molecule SCD inhibitor, diminishes NANOG-positive stem cells in induced pluripotent stem cells (iPS) and prevents tumorigenicity of iPS derivatives (iPSD) [148], suggesting that lipid desaturation is also required for NANOG-mediated CSCs generation. It is worthy to note that PluriSin#1 preferentially induces NANOG-positive stem cell apoptosis but has little effect on differentiated cardiomyocytes derived from iPS, showing a promising clinical application for cancer therapy.

### SREBP1

**Fig. 4:**
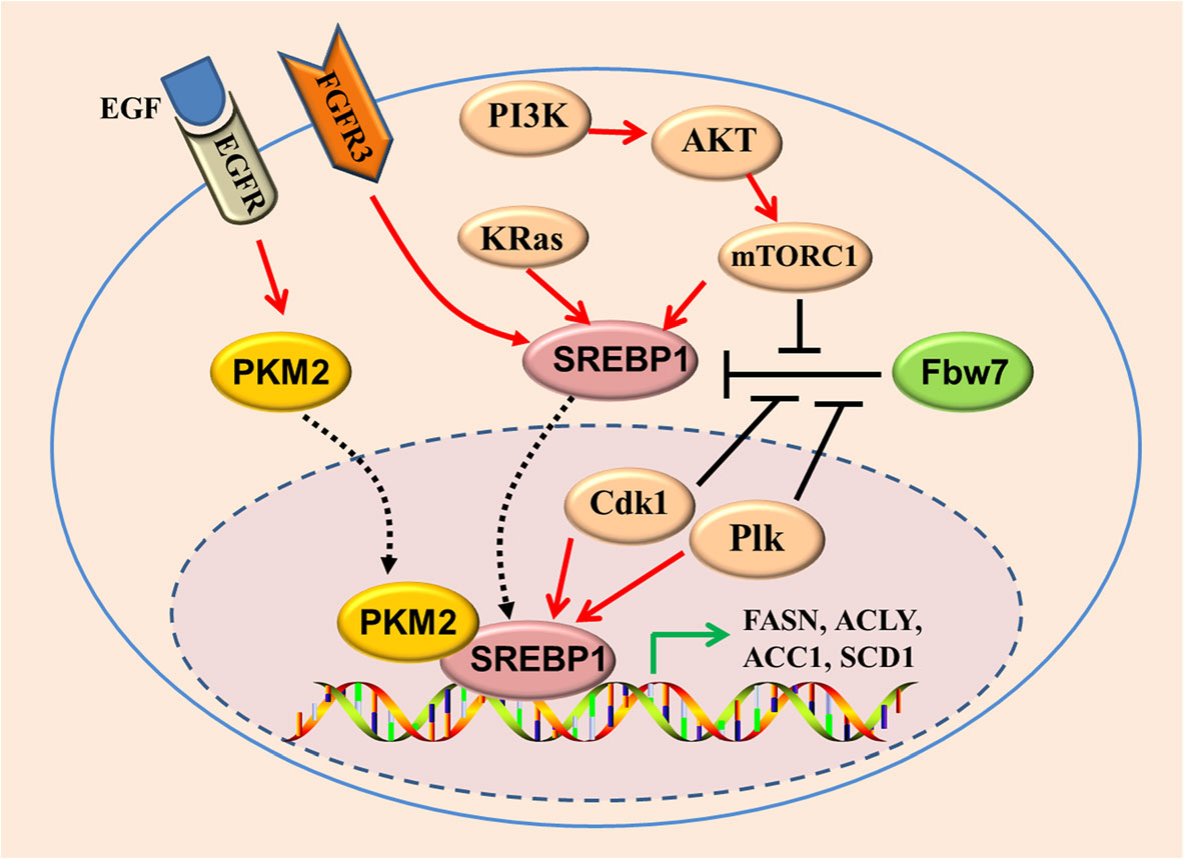
Interaction between oncogenic signaling and lipid desaturation in CSCs. Oncogenic activation of K-RAS, PI3K/AKT/mTOR signaling stimulates de novo lipogenesis via upregulation of SREBP1. Increase of SCD1 expression and lipid desaturation by NANOG or oncogenic signaling in CSCs or TICs reciprocally amplify NF-κB, Wnt/β-catenin, and Yap activation. Activation of JAK/STAT3 promotes CPT1B expression and activates the FAO pathway, which in turn contributes to Src oncoprotein activation. SREBP1, sterol regulatory element-binding protein-1

SREBP1, a master transcriptional regulator of lipogenesis, belongs to the SREBP transcription factor family and plays an important role in fatty acid and cholesterol biosynthesis [149]. SREBP1 is required to maintain lipogenesis under lipid- and oxygen-deprived conditions. Several lipogenesis enzymes are directly regulated by SREBP1, including ACLY, ACC1, and FASN [104] (Fig. 4). SREBP1 overexpression is observed in various human cancers and promotes tumor growth [150–154]. SREBP1 is also upregulated in BCSCs, supporting its stem cell behavior [104]. Activation of SREBP1 and SREBP2 is required for oncogenic PI3K (H1047R)- and K-Ras (G12 V)-stimulated de novo lipid synthesis and breast epithelial cell growth [155] (Fig. 4). In addition to promoting lipogenesis, SREBP1 contributes to generation of mono-unsaturated fatty acids by inducing SCD1 expression [156, 157] (Fig. 4). Inhibition of SREBP1 significantly blocks spheroid growth in glioblastoma [157]. SREBP1 protein is stabilized upon sequential phosphorylation by mitotic kinase Cdk1 and Plk1 during mitosis, blocking binding between the ubiquitin ligase Fbw7 and SREBP1 and attenuating SREBP1 degradation [158–160] (Fig. 4). In addition, nuclear accumulation of mature SREBP1 is promoted by PI3-kinase/Akt/target of rapamycin (mTOR)C1 signaling [161] (Fig. 4). Activation of EGFR signaling induces nuclear translocation of pyruvate kinase M2 (PKM2) [162], a key enzyme in Warburg effect [163]. A latest study unveiled that nuclear PKM2 physically interacts with SREBP1 and stimulates lipid biosynthesis through stabilizing SREBP-1 protein(Fig. 4), providing further evidence to show the crosstalk between glycolysis and fatty acids metabolism [164].

### MYC

MYC is overexpressed in various human cancers [165], and its potential to reprogram the cellular metabolism in cancer is well-recognized [166–168]. Importantly, the role of MYC in metabolic reprogramming has been confirmed in vivo. Though MYC is not necessary for hepatoblastoma development, depletion of MYC delays tumor progression through reducing fatty acid transporter CD36 expression, with a concomitant decrease in LDs accumulation and FAO levels [169]. Inhibition of FAO significantly delays constitutively active MYC-driven lymphoma development in a transgenic model [170, 171], whereas increase in FAO facilitates the tricarboxylic acid (TCA) cycle and ATP production in MYC-overexpressing TNBC [172]. It is worthy to note that inhibition of FAO by etomoxir in a MYC^high^ TNBC patient-derived xenograft (PDX) reduces the energy metabolism and inhibits tumorigenesis but has little effect on a MYC^low^ TNBC PDX model, suggesting a therapeutic potential in MYC-overexpressing TNBC tumors [129].

### SCD

SCD1 and SCD2 are two major isoforms of SCDs. Deletion of SCD1, the most abundant desaturase, decreases cardiac FFA and ceramide content in mice by reducing lipogenesis and activating lipolysis [173]. SCD2 expression is up-regulated to compensate SCD1 deficiency in mice liver [174]. Increased SCD1 expression has been observed in CSCs from ovarian, lung, breast cancer, and HCC [90, 109, 118, 175, 176]. In early stages of lung ADC, SCD1 is co-expressed with CSCs markers (CD133, CD24, CD44, and SOX2) [177]. High expression levels of SCD1 are tightly associated with disease progression and unfavorable clinical outcomes in lung cancer [177], HCC [118, 176], and breast cancer [105]. SCD1 expression is also negatively correlated with tumor differentiation in human HCC [178].

SCD1 is pivotal for CSCs/TIC generation and stemness maintenance [90, 118, 119, 175]. For example, silencing SCD1 in MCF10A cells significantly reduces mammosphere formation and the number of CD44^+^/CD24^−^ cells [109]. Genetic disruption of either SCD1 or SCD2 similarly inhibits TIC self-renewal and prevents experimental HCC formation induced by chemical carcinogens in mice [118]. However, it’s remain elusive whether SCD1 overexpression enhances stemness in non-stem cancer cells. Surprisingly, SCD1 is decreased in LSCs and plays a tumor-suppressive role in chronic myeloid leukemia [179], indicating that SCD1 function is context-dependent.

SCD1 expression is regulated by distinct oncogenic signaling pathways. SCD1 levels are transcriptionally and translationally controlled by mammalian mTOR signaling [180] (Fig. 2). In rodent HSCs and TICs, SCD1 expression is induced by Wnt-β-catenin signaling and reciprocally stabilizes the β-catenin protein [118] (Fig. 2). Li Junjie et al. demonstrated that NF-kB p65 binds to the SCD1 gene promoter (− 215 to − 206 and + 79 to + 88 bp) and induces its expression in ovarian cancer [90] (Fig. 2). Furthermore, SCD1 is a target of NANOG and is required for NANOG-mediated TIC generation [60] (Fig. 2). In breast cancer cells, SCD1 expression is induced by 17β-estradiol [181]. SCD1 expression is also induced by cancer-associated fibroblasts-released factors and promotes breast cancer cells migration, thus linking tumor microenvironment to metabolic reprogramming of CSCs [182]. Fibroblast growth factor receptor 3 (FGFR3) also stimulates SCD1 expression to accelerate tumor growth via activating SREBP1 in bladder cancers [183] (Fig. 4).

Genetic or pharmacological inhibition of SCD1 exerts a powerful anti-CSCs effect in various cancer types, including lung [175, 177], colon CSCs [184], ovarian [90], breast [109], and liver cancers [118, 178]. Silencing SCD1 or inhibiting its activity with betulinic acid (BetA) leads to rapid cell death in colon CSCs [184]. ROS generation may largely contribute to apoptosis after SCD1 inhibition [185]. Furtehr more, SCD1 activity is required for autophagosome formation [186]. SCD1 inhibition triggers cell death of pancreatic β-cells due to impairment of autophagy [187]. Nevertheless, the role of autophagy in SCD1 inhibition-induced cell death is controversial because pharmacological inhibition of SCD1 induces autophagic cell death via stimulating AMPK signaling [188]. SCD1 inhibition also induces liver TIC differentiation via the ER stress-induced unfolded protein response [176]. Importantly, inhibition of SCD1 overcomes drug resistance of lung adenocarcinoma CSCs to cisplatin [177] and liver TICs to chemotherapeutic drugs [176, 178], making it a desirable approach for novel combined therapeutic strategies.

### FASN and ACSVL3

FASN is a key enzyme in lipogenesis. It is highly expressed in patient-derived GSCs but markedly decreased after differentiation. Treatment of GSCs with cerulenin, a pharmacological inhibitor of FASN, leads to reduction of de novo lipogenesis and loss of stemness [103]. Overexpression of very long-chain acyl-CoA synthetase 3 (ACSVL3) has been demonstrated in lung cancer and glioma [189, 190]. Activation of both oncogenic receptor tyrosine kinases (RTK) c-Met and EGFR contributes to increased ACSVL3 levels in glioma cells, whereas silencing ACSVL3 leads to de-activation of Akt signaling [189]. Recently, Sun et al. demonstrated that ACSVL3 is implicated in GSCs maintenance and tumor initiation capacity. In addition, knockdown of ACSVL3 in neurosphere cells impairs its self-renewal and induces differentiation [191].

### CD36

CD36 is a scavenging receptor that is enriched in CSCs [124, 125] (Fig. 1). It has been shown that CD36, ITGA6, and CD133 are co-expressed in glioblastoma, and CD36 may be used to functionally distinguish CSCs from non-CSCs [125]. Otherwise, CD36 is highly expressed in metastasis-initiating cells (CD44^bright^ dye^+^) of OSCC cells compared to its CD44^bright^ dye^−^ counterpart. CD36(−) cells lose the ability to form a single lymph node metastasis, whereas CD36(+) cells develop more lymph node metastases than their parental cells. However, both CD36(+) and CD36(−) cells efficiently form oral lesions and primary tumors when orally inoculated into NSG mice, highlighting it’s distinct role of CD36(+) cells in metastasis. CD36(+)CD44^bright^ cells have higher expression levels of key enzymes involved in FAO (ACADVL, ACADM, and HADHA). Blocking lipid uptake with anti-CD36 antibodies in metastasis-initiating cells inoculated in mice successfully prevents metastasis initiation but not primary tumor formation [126]. In addition, loss of CD36 significantly sensitizes LSCs to chemotherapy and impairs tumorigenicity in mice [124]. However, challenging the accepted notion that chemo-resistant cells are LSCs, Farge et al. demonstrated that cytarabine (AraC)-resistant cells are neither LSCs nor quiescent cells, although AraC-resistant cells exhibit high levels of CD36 expression and FAO activity [192].

### CPT1A and CPT1B

Mitochondrial FAO is initiated with the transfer of long-chain fatty acids from the cytosol into the mitochondrial matrix by CPT1 and CPT2. CPT1 (also named CPT1A) is a rate-limiting enzyme in FAO and is located in the outer mitochondrial membrane (Fig. 1), whereas CPT2 is located in the inner mitochondrial membrane. CPT1A is overexpressed in prostate cancer and is associated with a high tumor grade [193, 194]. High expression levels of CPT1A predict unfavorable clinical outcomes in AML [195] and ovarian cancer [196]. Genetic or pharmacological inhibition of CPT1A exerts anti-tumor activity in prostate cancer [193], melanoma [197], breast [198], and ovarian cancer [196]. CPT1A is required for stem cell maintenance in neural stem/progenitor cells (NSPCs) [138]. CPT1A-dependent FAO flux is high in quiescent NSPCs but decreases in proliferating NSPCs. Reduced FAO flux triggers NSPC differentiation and loss of pluripotency [139]. CPT1B is one of the three isoforms of CPT1. Recently, Wang et al. demonstrated the role of JAK/STAT3 signaling in the regulation of BCSCs and cancer chemoresistance through promoting CPT1B expression and FAO in BCSCs. Blocking JAK/STAT3 signaling inhibits the self-renewal of BCSCs and re-sensitizes them to chemotherapy [89].

## Targeting lipid metabolism as novel therapeutic strategies against CSCs

CSCs are resistant to most traditional treatments. However, their dependency on lipid metabolism for proliferation and survival offers an Achilles heel for the elimination of these cells. Targeted clearance of CSCs could be achieved by intervening in different aspects of fatty acid metabolism such as lipogenesis, lipid uptake, lipid desaturation, and FAO. Due to the high costs and risk to discover and develop novel therapeutic agents, therapeutic strategies of drug repositioning for difficult-to-cure diseases treatment gain increasing attentions [40]. For instance, terfenadine, a “conventional” agents used to auto-immune disorders such as allergic dermatitis, has been demonstrated to reduce VEGF secretion from mast cells resided in the hypoxic microenvironment, and exerts great potential to kill melanoma cells via ROS-mediated apoptosis and autophagy [199, 200].

### Targeting lipogenesis

FASN is the most targetable among lipogenesis genes. Several FASN inhibitors display anti-tumor activity in preclinical cancer models (Table 1). Most importantly, some inhibitors show selective activity against CSCs rather than bulk tumor cells. Strikingly, Orlistat, a FDA-approved anti-obesity drug targeting FASN, exerts a potent anti-tumor activity in various cancers [201, 202]. Remarkably, inhibition of FASN by Orlistat in EGFR mutated NSCLC suppresses tumor growth in vitro and in vivo through reducing EGFR palmitoylation but inducing mutant EGFR ubiquitination and subsequent proteasomal degradation [203].

**Table 1:** Inhibitors of lipid enzymes involved in CSCS

Metabolism type	Drug	Targeting enzyme	Cancer type	Stage
Lipogenesis	Resveratrol	FASN	Breast cancer CSCs [104, 209]. Glioblastoma CSCs [210, 231]. Pancreatic CSCs [232]	Clinical Trial
	Cerulenin	FASN	Glioma CSCs [103], Pancreatic CSCs [91]	Pre-clinical
	Orlistat	FASN	NSCLC [203]	FDA-approved anti-obesity drug
Lipid uptake	CD36 antibody	CD36	OSCC [126]	Pre-clinical
	MTN	CD36	Glioblastoma CSCs [125]	Pre-clinical
FAO	Etomoxir	CPT1A	MYC-overexpressing TNBC [129], leukemia [219, 220]	Pre-clinical
	ST1326	CPT1A	Lymphoma [170], acute myeloid leukemia [221]	Pre-clinical
Lipid desaturation	SSI-4	SCD1	Liver CSCs [176],	Pre-clinical
	BetA	SCD1	CRC [184]	Pre-clinical
	PluriSin#1	SCD1	Teratomas [148]	
	MF-438	SCD1	Lung cancer CSCs [177]	Pre-clinical
	A939572	SCD1	CRC [185], clear cell renal cell carcinoma [233]	Pre-clinical
	Cay10566	SCD1	Breast Carcinoma [216]	Pre-clinical
	T-3764518	SCD1	CRC [234]	Pre-clinical

*OSCC* oral squamous cell carcinomas, *CRC* colorectal cancer, *TNBC* triple-negative breast cancer, *MTN* 2-methylthio-1,4-naphthoquinone, *FAO* fatty acid β-oxidation

#### Resveratrol

Resveratrol is a natural phytochemical compound extracted from grapes, red wine, berries, and peanuts(Table 1). Pre-clinical trials revealed that resveratrol exerts chemo-therapeutic and chemo-preventive effects against human cancers [204]. Further more, recent study demonstrated that resveratrol inhibits EMT and overcomes doxorubicin resistance in gastric cancer through modulating PTEN/Akt signaling pathway [205].. It has been shown that resveratrol at low dosage stimulates KEAP1/Nrf2 pathway and protects cells against oxidative agents, which may be the underlying mechanisms for it’s cancer chemoprevention function. However, high concentrations of resveratrol promotes ROS production leading to cell death [206–208]. Resveratrol suppresses lipogenesis and induces apoptosis in BCSCs by suppressing FASN expression [104, 209], making it attractive for clinical utilization. Resveratrol treatment inhibits GSCs proliferation at low doses and induces necrosis at higher doses but has no effect on normal NSCs behavior [210]. SIRT2 activation is required for resveratrol-mediated GSCs proliferation arrest but not for necrosis induced by high doses of resveratrol. Several clinical trials are currently underway [NCT00721877, NCT00920803, NCT00433576, and NCT00578396]. Pterostilbene, a dimethylated derivative of resveratrol with a higher bioavailability and is more lipophilic, exerts more potent suppressive activity against CSCs and cancer metastasis [211, 212].

#### Cerulenin

Cerulenin, a fungal metabolite, is another natural pharmacological inhibitor of FASN (Table 1). Cerulenin preferentially inhibits pancreatic CSCs proliferation compared to its parental cell [91]. It also inhibits glioma CSCs proliferation, migration and induces CSCs differentiation to glial cells [103]. The anti-tumor activity of cerulenin is enhanced by the combined use of oxaliplatin in human colon cancer cells [213]. Cerulenin also exerts an inhibitory effect on protein palmitoylation, thus affecting CD36 membrane trafficking [214].

### Targeting lipid desaturation

It is noteworthy that the SCD requirement for TIC generation was recently confirmed in a genetic mouse model [118]. High dependence on unsaturated fatty acids makes SCD a promising target to eradicate CSCs [215]. Several kinds of SCD1 inhibitors exhibit anti-tumor activity in pre-clinical cancer models (Table 1). SSI-4 is a novel SCD1 inhibitor that reverts sorafenib resistance in liver CSCs. SSI-4 display anti-tumor activity against liver CSCs without serious side effects in pre-clinical animal models [176]. Importantly, SSI-4 in combination with sorafenib show a maximal suppressive effect against tumorigenesis in a sorafenib-resistant PDTX model. Notably, the minimum dose of SSI-4 that has anti-tumor activity is approximately 10 mg/kg, a much lower concentration than that of other inhibitors such as A939572. MF-438 is another SCD1 inhibitor that reverts cisplatin resistance in lung CSCs [176]. A939572 and Cay10566 are also two widely used SCD1 inhibitors [173, 182, 185, 216]. A series of novel and potent SCD inhibitors are currently being developed [217]. Table 1 summarizes the potent SCD1 inhibitors used in pre-clinical stage.

### Targeting FAO

The dependency on FAO of CSCs makes it reasonable to target these cells using FAO inhibitors. Etomoxir is a specific inhibitor of mitochondrial CPT1A [218] (Table 1). Camarda et al. used a clinically relevant PDX model and found that etomoxir treatment markedly decreases ATP production and tumor growth in a MYC^high^ TNBC PDX model [129]. Etomoxir inhibits FAO in leukemia CSCs, thus suppressing cell proliferation and sensitizing human leukemia cells to apoptosis [124, 219, 220]. ST1326 is a CPT1A inhibitor that inhibits FAO and exerts cytotoxic activity against leukemia cell lines but not against normal CD34^+^ bone marrow cells [221] (Table 1). ST1326 treatment also prevents MYC-driven lymphomagenesis in a Eμ-myc transgenic mice model [170].

### Targeting lipid uptake

The scavenger receptor CD36 is enriched in CSCs and is responsible for extracellular lipid uptake [124–126]. Increased lipid uptake is observed in melanoma, glioblastoma, and leukemia CSCs, offering a promising avenue to develop novel therapeutic strategies [99, 123, 124]. Inhibition of CD36 by 2-methylthio-1,4-naphthoquinone treatment decreases self-renewal and induces apoptosis of CSCs in glioblastoma [125] (Table 1). Alternatively, CD36-neutralizing antibodies block extracellular lipid uptake of CSCs in OSCC and markedly inhibits tumor growth without side effects [126] (Table 1). Dependence on extracellular lipids also makes it reasonable to develop lipid nanoparticles as a Trojan horse to deliver drugs into CSCs. Many such strategies are being developed [222, 223]. For example, delivery of lipid nanoparticles containing miR-200c combined with paclitaxel (PTX) into breast CSCs exhibits profound anti-tumor activity [224].

## Conclusions

Recent advances in metabolomics have deepened our understanding of the contribution of metabolic reprogramming to tumorigenesis, which is now a well-recognized hallmark of cancer [225]. Alterations in lipid metabolism such as increase in fatty acid uptake, de novo lipogenesis, formation of LDs, FAO, and lipid desaturation are intensively involved in CSCs generation and stemness maintenance. Fatty acid metabolism not only satisfies the energy demands and biomass production of CSCs, but also contributes to the activation of several important oncogenic signaling pathways, including Wnt/β-catenin and Hippo/YAP signaling. Targeting key players of fatty acids metabolism shows promising to anti-CSCs and tumor prevention effects.

Although targeting the cell metabolism provides promising opportunities for eliminating CSCs, we have to face the dilemma of heterogeneity and metabolic plasticity of these cells [226–229]. CSCs and tumor cells may adapt its metabolic profile based on nutrients availability. For example, when the Warburg effect is reversed with cetuximab, HNSCC cells express high levels of ACC, which rewires cancer metabolism from glycolysis to lipogenesis to support energy demands and proliferation [230]. Although increased lipogenesis has been well documented in CSCs from various cancer types, most chemical compounds targeting FASN does not show a therapeutic efficacy in pre-clinical cancer models and only one FASN inhibitor has entered clinical trials. Owing to the metabolic flexibility of CSCs, it is difficult to effectively eliminate these cells by targeting a single metabolic pathway. A great challenge is to develop strategies to synergistically target multiple metabolic pathways in CSCs. An additional challenge comes from the metabolic similarities between CSCs and normal stem cells. For example, mitochondrial FAO is essential for NANOG-driven HCC TIC generation [60], but also contributes to expansion of normal HSCs and NSCs [137, 138]. Thus, the side effects of FAO inhibitors on normal HSCs and NSCs will have to be considered.

## Abbreviations

ACADM: Acyl-CoA dehydrogenase medium chain
ALDH1: Aldehyde dehydrogenase isoform 1
CoA: Acetyl coenzyme A
CSCs: Cancer stem cells
EGFR: Epidermal growth factor receptor
EMT: Epithelial-mesenchymal transition
FAO: Fatty acid β-oxidation
HADHA: Hydroxyacyl-CoA dehydrogenase trifunctional multienzyme complex subunit alpha
HCC: Hepatocellular carcinoma
HIF1: Hypoxia-inducible factor 1
HNSCC: Head and neck squamous cell carcinomas
TBNC: Triple negative breast cancer
TICs: Tumor initiating cells
VEGF: Vascular endothelial growth factor
