# Correction to: Emerging role of lipid metabolism alterations in Cancer stem cells

**DOI:** 10.1186/s13046-018-0826-z

**Published:** 2018-07-16

**Authors:** Mei Yi, Junjun Li, Shengnan Chen, Jing Cai, Yuanyuan Ban, Qian Peng, Ying Zhou, Zhaoyang Zeng, Shuping Peng, Xiaoling Li, Wei Xiong, Guiyuan Li, Bo Xiang

**Affiliations:** 1 Hunan Provincial Cancer Hospital and Cancer Hospital Affiliated to Xiangya Medical School, The Central South University, Changsha, 410013 Hunan China; 2 Department of Dermatology, Xiangya hospital of Central South University, Changsha, 410008 China; 3 Cancer Research Institute, Xiangya School of Medicine, Central South University, Changsha, 410078 China

## Correction

In the publication of this article [1], there is an error in the Figure caption of Figs. 2, 3 and 4. This has now been included in this correction. The authors declare that these corrections do not change the results or conclusions of this paper.

**Fig. 2:**
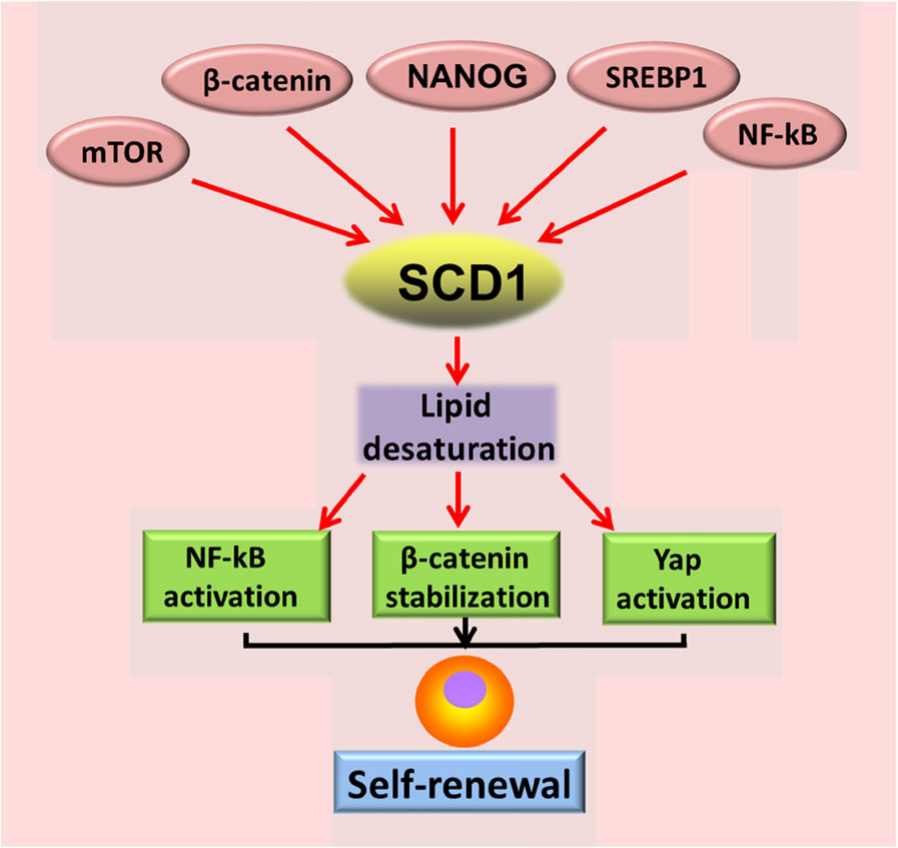
Interaction between oncogenic signaling and lipid desaturation in CSCs. Activation of NANOG, SREBP1 and oncogenic signaling such as Wnt/β-catenin, mTOR and NF-κB signaling stimulate lipid desaturation via induction of SCD1 expression. Increase of lipid desaturation reciprocally amplify NF-κB, Wnt/β-catenin, and Yap activation, which contribute to the self-renewal of CSCs or TICs. SREBP1, sterol regulatory element-binding protein-1

**Fig. 3:**
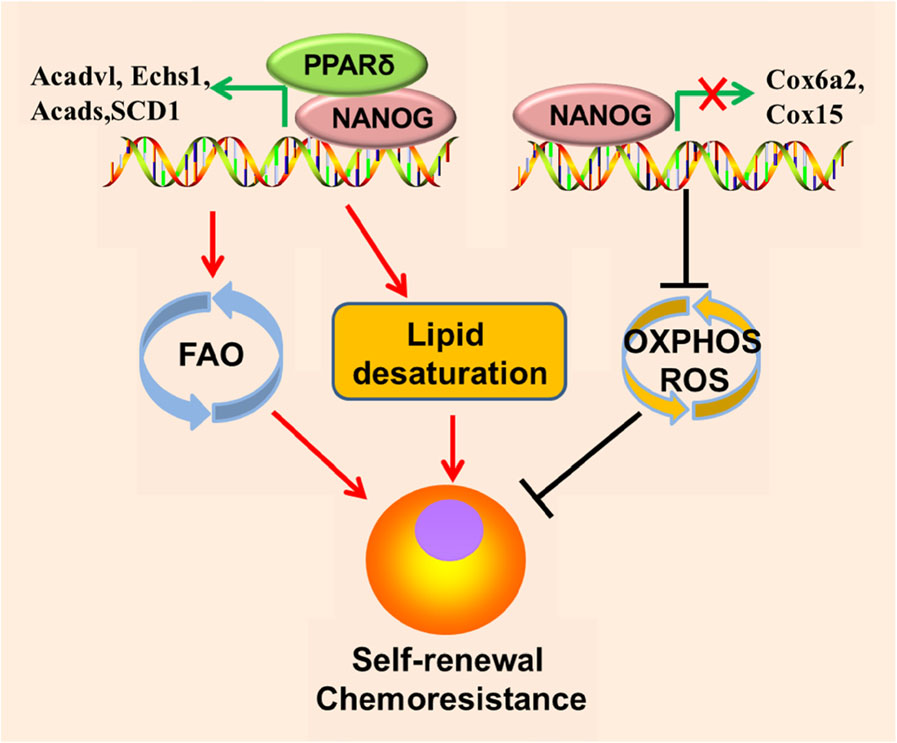
NANOG mediated metabolic reprogramming contributes to CSCs self-renewal and chemoresistance. NANOG binding on FAO genes (Acadvl, Echs1, and Acads) promoters stimulates it’s transcription but exerts opposite effect on OXPHOS genes(Cox6a2 and Cox15) transcription, leading to metabolic switch from OXPHOS to FAO and less ROS production in CSCs/TICs. NANOG also promotes lipid desaturation via up-regulating SCD1 expression. OXPHOS, oxidative phosphorylation; FAO, fatty acid oxidation

**Fig. 4:**
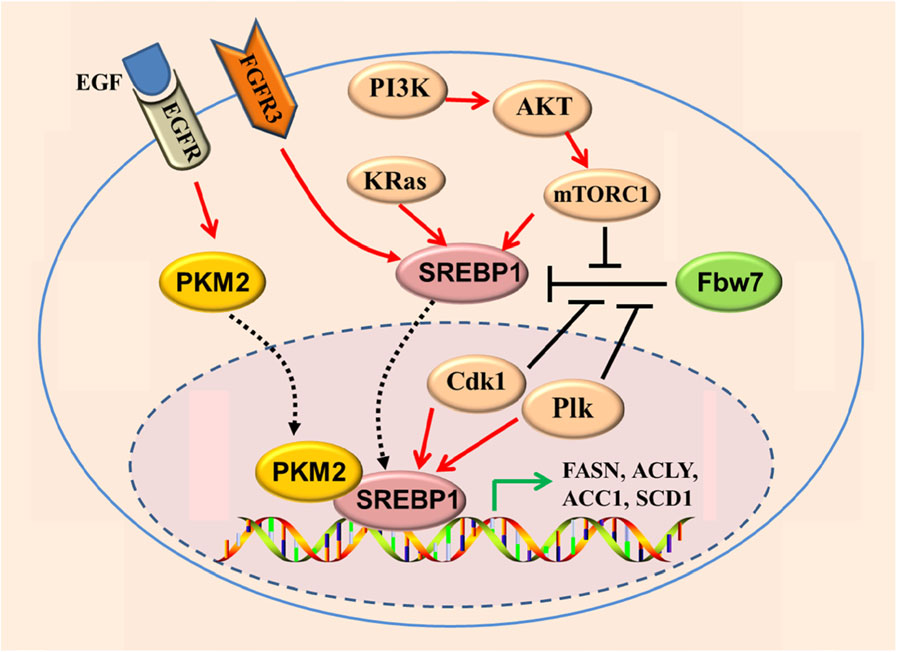
Regulation of SREBP1 and lipid metabolism by oncogenic signaling in CSCs. Oncogenic PI3K (H1047R)- and K-Ras (G12V) activates SREBP1 and SREBP2 to support de novo lipid synthesis and cell growth. The mTOR signaling regulates SREBP1 level through both transcriptional or translational mechanisms. Activation of PI3K/AKT/mTORC1 signaling pathway or FGFR3 leads to stabilization of SREBP1 protein and promotes SREBP1 translocation to nucleus. Mitotic kinase Cdk1 and Plk1 physically interact with nuclear SREBP1 protein. Sequentially phosphorylation of SREBP1 by Cdk1 and Plk1 blocks binding between the ubiquitin ligase Fbw7 and SREBP1 and attenuates SREBP1 degradation. Upon EGFR signaling activation, the nuclear form of PKM2 physically interacts with SREBP1, activating SREBP target gene expression and lipid biosynthesis

The error: In the initially published version of this article, the caption for Figs. 2, 3 and 4 were incorrectly presented due to a mistake.

The correct captions for Figs. 2, 3 and 4 are given hereafter:
